# Unraveling the Nexus: The Role of Collapsin Response Mediator Protein 2 Phosphorylation in Neurodegeneration and Neuroregeneration

**DOI:** 10.1007/s12017-024-08814-0

**Published:** 2024-11-12

**Authors:** Yuebing Wang, Toshio Ohshima

**Affiliations:** 1 Department of Life Science and Medical Bioscience, Waseda University, Shinjuku-Ku, Tokyo, 162-8480 Japan; 2 Laboratory for Molecular Brain Science, Department of Life Science and Medical Bioscience, Waseda University, 2-2 Wakamatsu-Cho, Shinjuku-Ku, Tokyo, 162-8480 Japan

**Keywords:** Neurodegeneration, Neuroregeneration, CRMP, Phosphorylation

## Abstract

Neurodegenerative disease characterized by the progressive damage of the nervous system, and neuropathies caused by the neuronal injury are both led to substantial impairments in neural function and quality of life among geriatric populations. Recovery from nerve damage and neurodegenerative diseases present a significant challenge, as the central nervous system (CNS) has limited capacity for self-repair. Investigating mechanism of neurodegeneration and regeneration is essential for advancing our understanding and development of effective therapies for nerve damage and degenerative conditions, which can significantly enhance patient outcomes. Collapsin response mediator protein 2 (CRMP2) was first identified as a key mediator of axonal growth and guidance is essential for neurogenesis and neuroregeneration. Phosphorylation as a primary modification approach of CRMP2 facilitates its involvement in numerous physiological processes, including axonal guidance, neuroplasticity, and cytoskeleton dynamics. Prior research on CRMP2 phosphorylation has elucidated its involvement in the mechanisms of neurodegenerative diseases and nerve damage. Pharmacological and genetic interventions that alter CRMP2 phosphorylation have shown the potential to influence neurodegenerative diseases and promote nerve regeneration. Even with decades of research delving into the intricacies of CRMP2 phosphorylation, there remains a scarcity of comprehensive literature reviews addressing this topic. This absence of synthesis and integration of findings hampers the field’s progress by preventing a holistic understanding of CRMP2’s implications in neurobiology, thereby impeding potential advancements in clinical treatments and interventions. This review intends to compile investigations focused on the role of CRMP2 phosphorylation in both neurodegenerative disease models and injury models to summarizing impacts and offer novel insight for clinical therapies.

## Overview

Neurogenesis, defined as the birth of new neurons in the mammalian brain, is characterized by heightened activity in the early years of life, followed by a gradual decline with aging. This process is crucial for brain development and plasticity, and plays a significant role in learning and memory. The regeneration of the central nervous system (CNS) following damage by injury or disease, involves the restoration and replacement of nerve cells. For several centuries, the prevailing notion has been that the mature central nervous system lacks an inherent capability for regeneration following injury. This long-standing assumption was subsequently challenged by the identification of neural and glial precursor cells within the adult brain whose post-injury proliferative potential defied prior conventions (Laywell et al., 2000). In contrast to lower organisms that exhibit robust neural regeneration, the capacity for neural cell regeneration in highly evolved organisms, including humans, is severely limited. Conversely, lower organisms, such as amphibians and fish, show remarkable CNS regeneration capacity. For example, zebrafish can regenerate their spinal cord after injury, a capability largely absent in mammals (Cigliola et al., 2020; Ferreira et al., 2012). This constraint presents a formidable obstacle in neurological injury and disease treatment. The limited regenerative capacity of humans is partly due to the complex architecture and specialized functions of the mammalian CNS. Additionally, inhibitory factors in the mammalian CNS, such as glial scars and myelin-associated inhibitors, restrict neural regeneration. The growth cone, a dynamic structure from the tip of growing nerve cells, is important for neural development and regeneration. These structures possess the ability to sense and respond to guidance cues in the extracellular matrix, which direct their growth towards target cells and tissues. The intricate process of axonal guidance involves various molecular signals, including attractive and repulsive cues, which ensure the precise wiring of the nervous system. Understanding the mechanisms underlying growth cone navigation and neural regeneration further holds significant promise for the development of therapeutic strategies to enhance CNS repair in humans.

The collapsin response mediator protein (CRMP) family plays a pivotal role in growth cone formation and dynamic regulation. The CRMP family consists of five main members: CRMP1, CRMP2, CRMP3, CRMP4, and CRMP5, each of which plays a unique role in neuronal development and function. Additionally, the CRMP family of proteins is involved in neuronal development, inflammation, neurodegeneration, and regeneration following injury (Tobe et al., 2017; Tsutiya et al., 2017). CRMP2, which was initially identified as CRMP-62, is a pioneering member of the CRMP family (Nakamura et al., 2020). It is recognized as a pivotal mediator protein involved in axonal guidance and response to collapsin. CRMP2 facilitates the transport of tubulin dimers and other cargo essential for axonal elongation, thus contributing to its role in axonal outgrowth. The role of DPYSL2, encoding Collapsin Response Mediator Protein 2 (CRMP2), in CNS injury and neurodegenerative diseases has been increasingly elucidated through genome-wide association studies (GWAS). GWAS data have uncovered several significant single nucleotide polymorphisms (SNPs) within DPYSL2, such as rs2270641, which are linked to heightened risk for Alzheimer’s disease (AD), Parkinson’s disease (PD), and traumatic brain injury (TBI). These findings suggest that genetic variations in DPYSL2 may lead to dysfunctional CRMP2 signaling, contributing to the pathophysiology of neurodegeneration. These genetic associations suggest that DPYSL2 variants may affect CRMP2’s functional role in neuroprotection and recovery post-injury. Functional studies corroborate this, showing that aberrant phosphorylation of CRMP2, linked to DPYSL2 SNPs, leads to cytoskeletal instability, impaired axonal transport, and increased neuronal susceptibility to stress.

**Fig.1:**
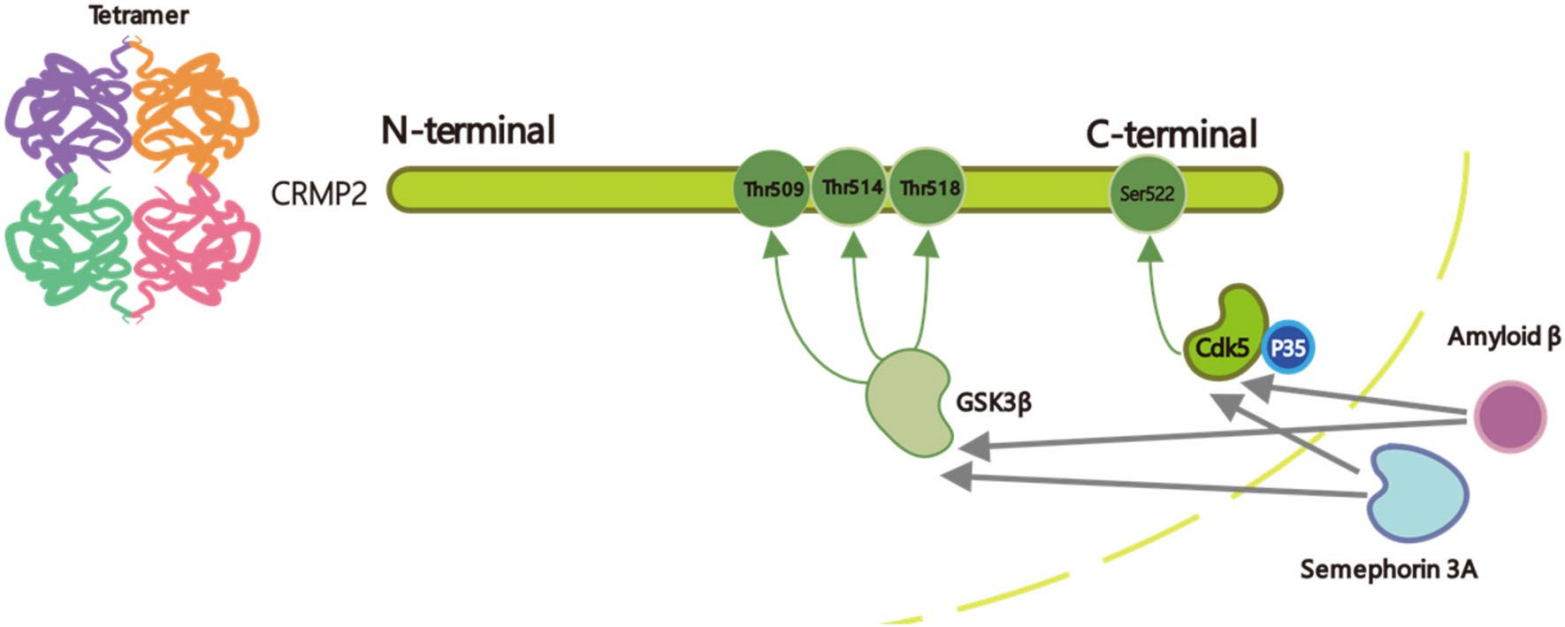
Diagrammatic representation of the CRMP2 phosphorylation process. CRMP2, which exists as a tetramer, undergoes sequential phosphorylation at the Thr509, Thr514, Thr518, and Ser522 residues. This phosphorylation cascade is initiated by the kinase Cdk5, in complex with its activator p35, which specifically targets Ser522. This event primes CRMP2 for subsequent phosphorylation at Thr509, Thr514, and Thr518 by GSK3β. The phosphorylation state of CRMP2 modulates its interaction with downstream signaling molecules, including Sema3A and Amyloid-β, which have been implicated in axonal guidance and neurodegeneration, respectively

Phosphorylated CRMP2, myelin-associated inhibitors, and semaphorin all congregate around scar tissue to suppress axonal growth and regeneration (Filbin, 2003; Nakamura et al., 2020). CRMP2 is functionally regulated by phosphorylation, Cyclin-Dependent Kinase 5 (Cdk5) phosphorylates CRMP2 at S522 to prime for sequential phosphorylation at T509, T514, and T518 by glycogen kinase 3β (GSK3β) (Fig. 1A). This sequential phosphorylation is crucial for modulating CRMP2 activity, with non-phosphorylated S522 preventing GSK3β from acting on other sites. In other words, phosphorylation by GSK3β at these three sites does not occur when S522 is non-phosphorylated. This CRMP2 dural phosphorylation was found to be caused by semaphorin 3a (Ssema3A), with decreased microtubule binding (Uchida et al., 2005). Cdk5, which is vital for brain development during embryogenesis, serves a crucial kinase function in regulating various neuronal processes. It controls the neuronal cytoskeleton through phosphorylation of microtubule-associated proteins such as CRMP2. While its typical activity supports neuronal functions like axon guidance, synaptic plasticity, and signal transduction, abnormal Cdk5 activity leads to pathological changes in the microtubule network, driving neuronal dysfunction and degeneration (Shah & Lahiri, 2017). Additionally, T555 is phosphorylated by Rho kinase, while the phosphorylation of other tyrosine residues has also been observed (Nakamura et al., 2020). These phosphorylation events collectively regulate the interactions of CRMP2 with microtubules and other cytoskeletal components. The mechanism of regeneration has been investigated by suppressing the phosphorylation of CRMP2. The generation of CRMP2 knock-in (CRMP2 KI) mice has further provided a solution to investigate neurodegenerative diseases and repair after damage. In this review, we discuss the role of CRMP2 phosphorylation suppression in the development of neurodegenerative diseases and regeneration after injury. Experimental approaches using CRMP2 KI mice have shown promising results in enhancing axonal regeneration and functional recovery post-injury. The inhibition approach was not only a genetic modification, but also involved the determination of various CRMP2 phosphorylation inhibitors.

The study of CRMP2 phosphorylation encompasses both the process and the inhibition of phosphorylation. In this paper, two compounds commonly cited in prior research were selected as representatives to review and summarize the effects of inhibiting CRMP2 phosphorylation in neurodegenerative diseases and neural injuries. Compounds known to inhibit CRMP2S522 phosphorylation include huperzine A and naringenin. Huperzine A (HupA) is a naturally occurring sesquiterpene alkaloid primarily extracted from plants of the Huperzia genus, such as Huperzia serrata. Huperzia serrata has long been used in traditional Chinese medicine for its cognitive-enhancing effects. It also serves as a potent, selective, and reversible acetylcholinesterase (AChE) inhibitor leading to increased acetylcholine levels and improved synaptic function, making it a therapeutic candidate for neurodegenerative diseases (H. Zhang, 2012). However, recent studies have revealed that HupA’s neuroprotective benefits extend beyond its cholinergic activity. One such mechanism involves the inhibition of collapsin response mediator protein 2 (CRMP2) phosphorylation, which is crucial for maintaining axonal integrity. A study of amphetamine-induced hyperlocomotion model firstly demonstrated that HupA effectively inhibits CRMP2 phosphorylation through its interaction with GSK3β (Zhao et al., 2020) HupA’s ability to prevent this pathological phosphorylation suggests a broader neuroprotective role, offering therapeutic potential in diseases where axonal degradation is a central feature. Naringenin (NAR) is a naturally occurring flavonoid predominantly found in citrus fruits, including grapefruit, oranges, and lemons. It is well-known for its strong anti-inflammatory and antioxidant properties, which confer potential health benefits (Jiang et al., 2022). NAR can easily cross the blood–brain barrier and exerts protective effects on neuronal health through various mechanisms, one of which is its ability to modulate intracellular signaling pathways. NAR was proved with the specific effect on binds with CRMP2 to reduce its phosphorylation in a neurotherapeutic screening study via bioinformatics and computational tools (Khanna et al., 2020; Lawal et al., 2018). In this paper, we will delve into the multifaceted role of CRMP2 phosphorylation in the context of neurodegenerative disease and neuronal injury. Also, the inhibitory effect on CMRP2 phosphorylation inhibitors.

## From the Study of Neurogenesis to the Study of Neuroregeneration

Research interest in CRMP2 has remained steady, particularly from the perspectives of neurogenesis and development. This sustained interest is due to the pivotal role of CRMP2 in various neural processes, consistently demonstrated in experimental models. Analysis of CRMP2 gene knockdown/knockout mice and zebrafish has indicated its significant role in neural development and circuit formation. Indeed, these animal models have provided critical insights, revealing that CRMP2 is essential for the proper establishment of neural pathways and synaptic connectivity. It has further been reported that deficiencies in CRMP2 function may manifest as intellectual disabilities and other related symptoms in human patients (Suzuki et al., 2022). These deficiencies underscore the importance of CRMP2 in maintaining cognitive function and overall neurological health. The interaction between CRMP2 and other molecules, such as the binding of CRMP2 and KLC1, is essential for proper neural pathway connectivity, highlighting the crucial role of CRMP2 in the formation of forebrain commissures (Li et al., 2024). Sema3A and myelin-related factors have also been reported to inhibit nerve regeneration when nerve axons are damaged in the brain and spinal cord (De Winter et al., 2002; K. C. Wang et al., 2002). Inhibitory mechanisms complicate nerve injury treatment by creating an unfavorable environment for axonal repair and regrowth. Therefore, inhibition of CRMP2 phosphorylation may promote neuronal regeneration. Herein, we hypothesized that blocking the phosphorylation of CRMP2 could stabilize microtubules in nerve axons. Microtubule stabilization is vital for maintaining axonal integrity and ensuring effective nerve transmission and regeneration. Stabilization is also believed to be a key process that facilitates nerve regeneration. Consequently, we performed validation using the disease model outlined below.

## From Mouse Genetic Modification to Drug Effect Determination

In the CRMP2S522 mutant protein (CRMP2KI), the Cdk5 primary phosphorylation site S522 is substituted with the non-phosphorylated amino acid Ala (A); this mutation was been reported to be associated with abnormalities in dendritic patterning (Yamashita et al., 2012) (Fig. 1B). As such, this mutant is widely utilized as a CRMP2 phosphorylation inhibitory model in numbers of neural injury and neurodegenerative disease research. In clinical applications, the inhibition of CRMP2 phosphorylation through a pharmacological approach may have effects similar to those of CRMP2KI. Thus, approaches based on drug treatment to suppress CRMP2 phosphorylation have been widely studied for CNS injury and neurodegeneration. It is essential to consider that as many drugs exert additional effects beyond inhibiting CRMP2 phosphorylation, it is challenging to determine whether their impact on nerve regeneration is caused by this specific inhibition. For example, if an administered drug exhibits an anti-inflammatory effect, it could play a role in enhancing pathology. Identifying the precise mechanisms that contribute to the enhancement of the pathology could be complex. Clinically, even if it is a compound effect, it is important to determine the extent to which it improves pathological conditions such as nerve regeneration. Therefore, a comprehensive evaluation of drug effects, considering both specific and nonspecific actions, is important to understand their therapeutic potential. Concurrently, summarizing and comparing the roles of these two approaches within the same physiological process can guide future research and provide effective evidence for the development of future clinical therapies. Such comparative analyses are invaluable for elucidating the underlying mechanisms of action, and for optimizing treatment strategies for neurological disorders.

## Injury Models

### Spinal Cord Injury

CRMP2 is actively involved in the transport of tropomyosin receptor kinase B (TrkB), a specific receptor for brain-derived neurotrophic factor (BDNF), which is known to be modulated by phosphorylation (Yamashita & Goshima, 2012). In earlier studies (Nagai et al., 2016), comparisons made 24 h post-BDNF administration in the CRMP2 KI mouse model of spinal cord injury indicated that the dorsal root ganglion (DRG) originating from CRMP2 KI mice had increased axonal outgrowth compared to wild-type mice. CRMP2 regulates presynaptic excitatory neurotransmission, and may be crucial for controlling pathological pain (Yu et al., 2019). In addition, comparison of the phosphorylation of TrkB at the axon tip 10 min after BDNF addition revealed that the signal was stronger in CRMP2 KI-derived DRG neurons. In previous studies, T7-8 spinal cord-injured mice were observed for 4 weeks, and temperature sensation and lower-extremity motor function were evaluated using the hot plate method. Consistent with the outcomes from the DRG experiment, CRMP2 KI mice exhibited improved preservation of temperature sensation after spinal cord injury compared to wild-type mice. The Basso Mouse Scale (BMS) score, which is an evaluation score for lower-extremity motor function, also significantly improved over the 4 weeks after surgery. Histological examination further revealed axonal outgrowth extending beyond the injury site and an increase in the expression of growth-associated protein 43 (GAP43), a marker of growing axons. To verify whether these results could be replicated, we introduced a drug, Lanthionine ketimine ester (LKE), into the same model. LKE can cross the BBB by esterifying lanthionine ketimine (LK), which is present endogenously in the brain. LKE suppressed CRMP2 phosphorylation Mice injected intraperitoneally with 100 mg/kg LKE in PBS daily showed a superior recovery of motor function, as assessed by the BMS score after spinal cord injury, compared with mice injected intraperitoneally with the same dose of PBS (Kotaka et al., 2017).

### Optic Nerve Damage

The following studies focused on investigating optic nerve injury (ONI). Optic nerve crush (ONC) surgery is widely used to develop ONI models. In this model, the injured optic nerve undergoes Wallerian degeneration on the distal side. Wallerian degeneration is a process that occurs in the distal segment of a nerve fiber (axon) and its associated myelin sheath following nerve injury. During this process, the axon undergoes immediate changes in membrane potential, triggering numerous biochemical reactions, including alterations in the calcium ion concentration. Subsequently, the distal portion of the axon begins to disintegrate and the surrounding myelin sheath is degraded. Macrophages then infiltrate the injury site to clear debris. In some instances, nerve regeneration may occur, during which new axons extend from the neuron’s cell body to potentially reestablish connections with target tissues. Because Wallerian degeneration and the removal of damaged axons and myelin are essential for regeneration, suppression of Wallerian degeneration makes optic nerve regeneration difficult (Vargas & Barres, 2007). However, in the CRMP2 KI model, Wallerian degeneration was inhibited and regenerated axons were observed compared to the wild-type (Kondo et al., 2019). This was further evidenced by counting the labeled optic nerves after injection of a tracer into the eyeball, which showed few labeled axons beyond the injury site in wild-type mice, but many in CRMP2 KI mice. This observation aligns with the elevated levels of GAP43, an axonal regeneration marker, in the immunostaining and western blotting results. Sema3A induces M1-like microglial activation following ONC (Yun-Jia et al., 2021). Relatively weak microglial activation was observed in CRMP2 KI mice following ONC, compared to the same injury conditions in WT mice. Huperzine A, a traditional Chinese medicinal extract, has been shown to inhibit CRMP2 S522 phosphorylation in a normal tension glaucoma (NTG) model (Y. Wang et al., 2024a, 2024b), demonstrating significant efficacy in suppressing microglial activation after ONC surgery, indicating its potential as a novel therapeutic direction for addressing neuroinflammatory processes associated with optic nerve injury(Y. Wang et al., 2024a, 2024b).

## Neurodegenerative Disease Models

### Alzheimer’s Disease

AD is a progressive neurodegenerative disorder characterized by cognitive decline, memory loss, and behavioral changes that primarily affect the elderly population. The deposition of amyloid-beta (Aβ) plaques is a hallmark pathology of AD, which contributes to synaptic dysfunction and neuronal death. Hyperphosphorylation of the tau protein leads to the formation of neurofibrillary tangles, which disrupt the microtubule network and are strongly associated with AD progression (Bloom, 2014). Elucidating the relationship between Aβ and tau pathology is key to understanding the molecular mechanisms underlying AD, as both proteins are known to synergistically exacerbate neurodegeneration. Recently, CRMP2 was shown to be implicated in the pathophysiology of AD. Hyperphosphorylation of CRMP2 is considered a critical factor contributing to neurodegeneration. Research comparing the pathways leading to increased phosphorylation of CRMP2 with those responsible for tau hyperphosphorylation in AD proposed that common mechanisms, such as elevated kinase activity or diminished phosphatase function, may be involved (Soutar et al., 2009). Phosphorylated CRMP2 at Thr518, Thr514, and Thr509, mediated by GSK3β, have been found in neurofibrillary tangles in both human patients with AD and AD mouse models (Gu et al., 2000; Watamura et al., 2016). The rise in CRMP2 phosphorylation precedes pathological development in mouse models, implying that it may serve as an early indicator of AD (Soutar et al., 2009). Increased hippocampal phosphorylation of CRMP2 has also been found in wild-type mice following the intracerebroventricular injection of Aβ neurotoxic fragment (Isono et al., 2013). The interaction between CRMP2 and GluA, a subunit of the AMPA receptors, is inhibited by CRMP2 phosphorylation at S522 (Cheng et al., 2022). This suggests that the phosphorylation of CRMP2 induced by Cdk5 is important for the surface delivery of AMPA receptors in hippocampal neurons. Previous research has further revealed that the levels of CRMP2 phosphorylation and p35 protein are elevated in the cerebellum of APP/PS1 mice, while peroxisome proliferator-activated receptor γ (PPARγ) can normalize levels in the cerebellum (Toba et al., 2016).

Among the available CRMP2 S522 phosphorylation inhibitors, HupA is widely used in the treatment of AD, and as a neurotrophic supplement. Beyond its acetylcholinesterase inhibitory effects, prior studies have primarily explored its multifaceted neuroprotective mechanisms, including activation of the cholinergic system and direct action on mitochondria (H. Zhang, 2012). Biochemical analyses have confirmed that NAR is a bioactive metabolite that can be transferred across the blood–brain barrier. By combining drug affinity-responsive target stability with immunoprecipitation liquid chromatography/mass spectrometry analysis, CRMP2 was also identified as a target of NAR (Z. Yang et al., 2017).

### Normal Tension Glaucoma

Unlike optic nerve damage, which is rare, normal tension glaucoma (NTG) affects a large number of patients, and is especially prevalent among the elderly population. In general, increased intraocular pressure causes cell apoptosis in retinal ganglion cells (RGC, the origin of the optic nerve), leading to atrophy of the optic nerve, resulting in glaucoma. In NTG, RGC cell death and optic nerve atrophy occur, despite intraocular pressure being within the normal range. One of the main causes of RGC death in patients with NTG is glutamate excitotoxicity. Models mimicking NTG occurrence include those administered intraocular injections of the agonist N-methyl-D-aspartate (NMDA), and mice lacking the glutamate-aspartate transporter (GLAST), which eliminates glutamate. In both NMDA-injection and GLAST mutant models, CRMP2 KI mice demonstrated a slowing of RGC loss to some extent. Additionally, these two models show elevated CRMP2S522 phosphorylation in the RGC (Brahma et al., 2022). These findings suggest that CRMP2 phosphorylation is associated with the pathology of NTG.

HupA and Naringenin (NAR) were the two pilot natural compounds investigated for their ability to inhibit CRMP2 phosphorylation. Using the same model of NMDA-injection and GLAST mutation, HupA and NAR displayed protective effects against RGC loss and inner retinal layer thinning in histological analysis. Surprisingly, drug-treated GLAST ± mice exhibit showed decreases in CRMP2 phosphorylation at S522, which verifies our hypothesis that these two drugs function as CRMP2 phosphorylation inhibitors. Furthermore, the oxidative stress marker 4-Hydroxynonenal (4-HNE) was shown to be decreased in the drug-treated NTG model compared to that in un-treated (Y. Wang et al., 2024a, 2024b).

### α-Synucleinopathy

α-Synucleinopathies, a category of neurodegenerative disorders, are chiefly characterized by the pathological clumping of α-synuclein protein within neuronal and glial cells (Koga et al., 2021). A presynaptic neuronal protein, α-synuclein is highly expressed in the brain, playing important roles in synaptic vesicle trafficking, neurotransmitter release, and synaptic plasticity. Under pathological conditions, this protein undergoes conformational alterations, resulting in the formation of oligomers and fibrils that accumulate intracellularly as Lewy bodies and neurites (Bernal-Conde et al., 2020). These aggregates disrupt cellular homeostasis, triggering a cascade of neurotoxic events including mitochondrial dysfunction, impaired protein degradation, and neuroinflammation.

#### Parkinson’s Disease

Degenerative processes, which encompass axonal and dendritic structural disruption, abnormal axonal transport, release of extracellular factors, and inflammation, are all commonly regulated by the cytoskeleton. CRMP2 is a regulator of cytoskeletal protein assembly in neurons that functions by controlling axonal growth and neural circuit formation (Nagai et al., 2017). In PD, the loss of dopamine-producing cells in the mesencephalic substantia nigra leads to a decrease in the levels of dopamine in the striatum (to which the axons project), thus resulting in movement disorders such as bradykinesia and rigidity. However, it is thought that the pathology begins with the dopaminergic axons leading to the striatum. Therefore, we administered MPTP, which induces apoptosis of dopamine-producing cells in the mesencephalic substantia nigra, to CRMP2KI cells, and compared it with that of the wild-type. Overall, we found that dopaminergic nerve endings in the striatum are preserved in CRMP2KI mice compared to wild-type mice (Togashi et al., 2019). In addition, LKE administration suppressed cell death not only in nerve terminals, but also in dopaminergic neurons in the mesencephalic substantia nigra, while motor dysfunction was further improved(Togashi et al., 2020). The mechanism underlying this improvement may involve the anti-inflammatory action of LKE and its effects on autophagy.

#### Lewy Bodies Disease

Lewy bodies, which are formed from the accumulation of α-synuclein aggregates, have been pathologically characterized in midbrain substantia nigra dopamine neurons in patients with PD. Diffuse Lewy body disease (LBD), which is associated with cognitive decline, is classified as an α-synucleinopathy. In one study, the oral administration of LKE for 2 months to α-synuclein-overexpressing mice was shown to decrease the accumulation of α-synuclein in the hippocampus and cerebral cortex, and improved the fear conditioning test observed in α-synuclein-overexpressing mice (Yazawa et al., 2022). Building on this, we are currently conducting experiments to confirm whether the effect of LKE is mediated by the suppression of CRMP2 phosphorylation by mating mice overexpressing α-synuclein with CRMP2 KI. Increased phosphorylation of CRMP2 at the GSK3 phosphorylation site has been reported in the brains of patients with LBD. α-synuclein-overexpressing mouse brains showed a similar increase in phosphorylation, which was normalized by the administration of LKE(Yazawa et al., 2022). In addition, crossing CRMP2KI with SOD1G39A, a model of ALS, improved this pathology(Numata-Uematsu et al., 2019), suggesting that the suppression of CRMP2 phosphorylation may lead to pathological improvement in other neurodegenerative diseases.

#### Multiple Sclerosis

Multiple Sclerosis (MS) is a chronic, immune-mediated disorder of the central nervous system (CNS), characterized by demyelination, axonal damage, and neurodegeneration. It is an autoimmune disease in which the body’s immune system erroneously targets myelin, the protective sheath surrounding nerve fibers, leading to impaired neural communication. MS typically presents in young adults, particularly women, and manifests in various neurological symptoms, including motor dysfunction, sensory disturbances, and cognitive impairments. The disease course can vary between relapsing–remitting, primary progressive, or secondary progressive forms. While the etiology of MS remains unclear, it is believed to result from a complex interplay of genetic predispositions, environmental factors, and immunological triggers. Advances in diagnostic imaging, such as magnetic resonance imaging (MRI), have facilitated earlier and more accurate diagnoses. Current treatment strategies focus on immune modulation, with disease-modifying therapies aiming to reduce relapse rates and slow disease progression (Compston & Coles, 2008). MS involves demyelination and axonal degeneration in the central nervous system. Previous study reported that calcium influx induced by lesions activates the protease calpain, which cleaves CRMP2. This cleavage impairs axonal transport, contributing to axonal degeneration (Lee et al., 2014; J.-N. Zhang & Koch, 2017). Both calpain inhibition and CRMP2 overexpression were shown to protect proximal axons from acute degeneration. Specifically, CRMP2 phosphorylation site Thr555 was claimed to regulate axonal growth during the progression of experimental autoimmune encephalomyelitis in mouse models. The phosphorylation of CRMP-2 at Thr555 is dependent on the Nogo-66 receptor 1 (NgR1). Blocking the interaction between Nogo-A and NgR1 may reduce axonal degeneration and improve clinical outcomes in experimental autoimmune encephalomyelitis (Petratos et al., 2012). By modulating CRMP2 activity, it may be possible to both prevent axonal degeneration and promote axonal regeneration.

## Therapeutical Approaches

CRMP2 is increasingly recognized for its involvement in cancer conditions, with studies demonstrating its role in tumor cell migration, invasion, and metastasis across multiple cancer types (Shimada et al., 2014). CRMP2 serves as a diagnostic and prognostic biomarker in lung adenocarcinoma (Wu et al., 2021) and also a mediator of breast development and migration (Abu Rmaileh et al., 2022; Lin et al., 2020). These findings underscore the critical need to advance CRMP2 phosphorylation research from foundational studies to clinical applications, highlighting its potential for developing novel therapeutic strategies. CRMP2 has shown potential as a therapeutic target in various clinical treatments and therapies. Using vector-mediated gene transfer techniques such as adenoviruses or retroviruses can transfer normal CRMP2 gene into patient cells (Xu et al., 2024; Zhou et al., 2024). CRMP2 is considered a relatively specific therapeutic target due to its critical role in neuronal development and function. However, like many biological targets, there is potential for off-target effects, especially when using techniques like CRISPR-Cas9 for modifications (Zhu et al., 2024). Translating CRMP2 gene therapy into clinical applications entails overcoming various obstacles, such as achieving high target specificity to avoid unintended gene modifications, developing efficient delivery systems to maintain therapeutic gene expression, and mitigating immune reactions that may hinder the therapy’s effectiveness. The CRMP2 phosphorylation inhibitors such as HupA and NAR, frequently mentioned in this review, have been approved for human use and still hold significant potential in clinical applications. The development of new drugs targeting CRMP2 phosphorylation inhibition could provide a novel direction for future clinical trials (Zhao et al., 2020). Unavoidably, the clinical application of drugs targeting CRMP2 phosphorylation inhibition is accompanied by various side effects. Clinical studies have documented cholinergic adverse reactions such as hyperactivity, nasal obstruction, insomnia, anxiety, dizziness, thirst, and constipation, as well as cardiac ischemia and arrhythmia in patients treated with Huperzine A (G. Yang et al., 2013). Clinical studies on Naringenin have reported adverse effects including headaches, dizziness, and mild liver enzyme elevations, indicating the need for careful monitoring during therapeutic use (Salehi et al., 2019). In the development of future clinical treatments, it is crucial to bridge the research gap between animal models and human trials. Addressing the unknown mechanisms involved in inhibiting CRMP2 phosphorylation will further shorten the transition to clinical application.

## Conclusion

**Fig. 2:**
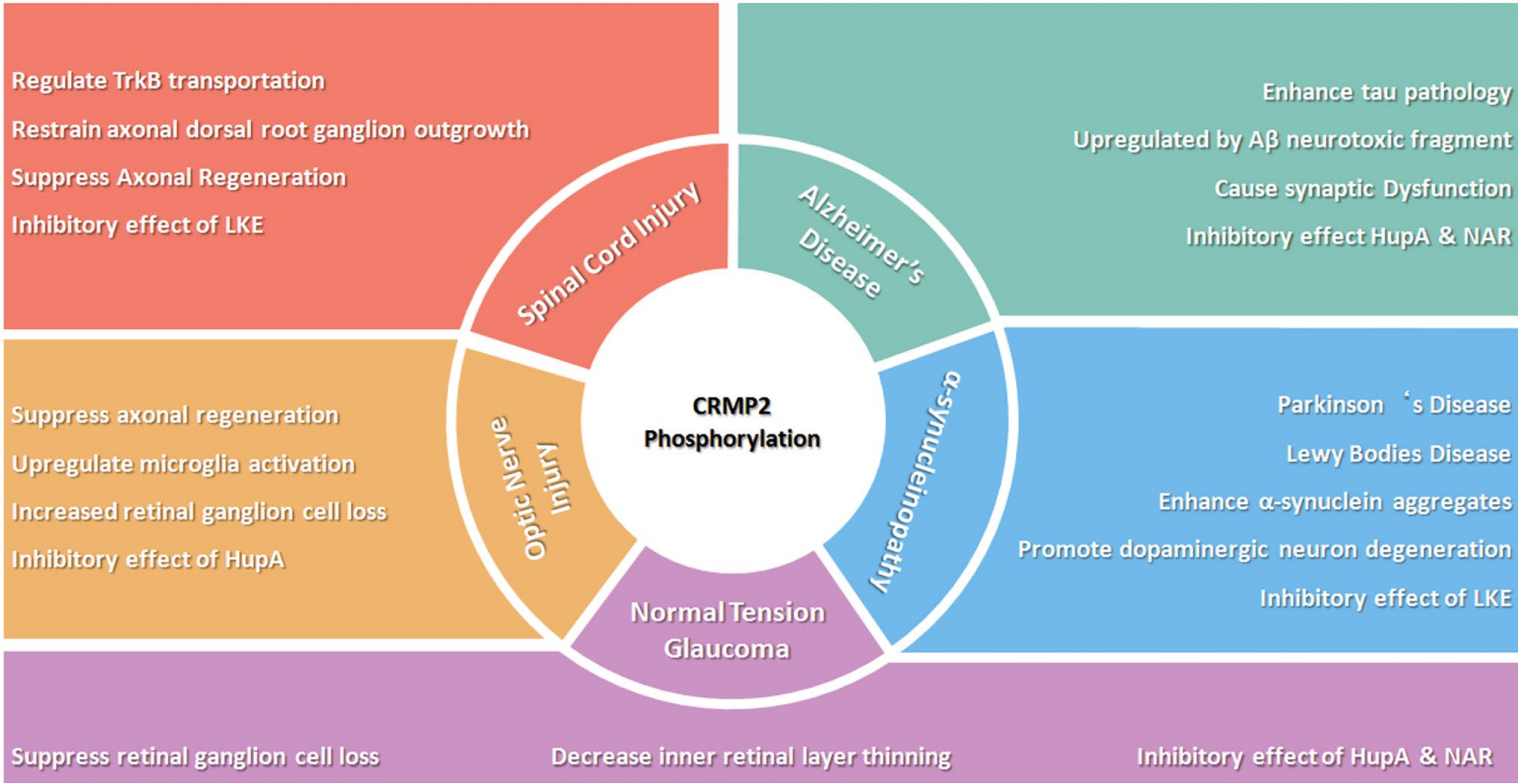
Summary of CRMP2 phosphorylation in injury model and neurodegenerative disease model. This diagram summarizes the effects of CRMP2 phosphorylation into two main domains: neural injury and neurodegenerative disease. The section on neural injury highlights the impact on spinal cord and optic nerve injuries, with implications for axonal growth, microglial activation, and therapeutic interventions. In the neurodegenerative disease section, its roles in Alzheimer’s disease, Parkinson’s disease, α-synucleinopathies, and normal tension glaucoma are outlined, focusing on the association with tau pathology, synaptic dysfunction, and potential therapeutic strategies targeting CRMP2

To conclude, this review seeks to summarize and distill research efforts on inhibiting CRMP2 phosphorylation via both pharmacological and genetic methods. The focus is predominantly on injury and neurodegenerative disease models (Fig. 2), aiming to offer fresh insights for future clinical treatment strategies. In our prior studies, we investigated whether the inhibition of CRMP2 phosphorylation contributes to neuronal regeneration in mouse models of disease. We plan to further investigate its usefulness as a method to promote CNS regeneration following injury. Inhibition of CRMP2 phosphorylation stabilizes neuronal axons and promotes microtubule polymerization, thereby contributing to axonal regeneration. Regarding the stabilization of microtubules, it has been reported that drugs such as Taxol, which are used to treat cancer, can improve axonal regeneration after spinal cord injury by inhibiting cell division, thus stabilizing microtubules. However, because the effects of microtubule stabilizers are not limited to nerve cells, there are concerns regarding potential strong side effects. In contrast, the inhibition of CRMP2 phosphorylation is considered an excellent therapeutic strategy for neurological diseases, as it contributes to the stabilization and polymerization of microtubules in a neuronal-specific manner. Screening for drugs that suppress CRMP2 phosphorylation has identified several candidates, including ropinirole, which is being studied as a therapeutic candidate for ALS. Existing research on CRMP2 phosphorylation mechanisms, regardless of whether it targets nerve injury or neurodegenerative disease models, ultimately aims to resolve the challenges associated with human neuropathy. Off-target effects in gene therapy and treatment-related side effects partially limit the feasibility of these studies in clinical translation. The gap between animal models and human clinical trials remains to be bridged. Meanwhile, deeper investigation into the finer mechanisms of CRMP2 phosphorylation remains warranted. Future research should specifically target the limitations mentioned above, such as studying the new phosphorylation site T555A of CRMP2, which enhances its interaction with CaV2.2 (Brittain et al., 2012)and regulates dendritic outgrowth in cerebellar Purkinje cells (Winkler et al., 2020). Overall, continued investigation of CRMP2 phosphorylation should enhance our understanding of neuronal biology and disease, ultimately contributing to the development of novel therapeutic strategies for neurodegenerative disorders and neuropathy.

## Data Availability

No datasets were generated or analysed during the current study.
